# Severe congenital kyphoscoliosis

**DOI:** 10.11604/pamj.2024.48.145.44441

**Published:** 2024-08-02

**Authors:** Souvik Sarkar, Pankaj Wagh

**Affiliations:** 1 Department of Respiratory Medicine, Datta Meghe Institute of Higher Education and Research, Wardha, Maharashtra, India

**Keywords:** Scoliosis, kyphosis, vertebral column defect, restrictive lung disease

## Image in medicine

A 6-year-old female child came to the outpatient department with complaints of exertional dyspnea and occasional cough. The child was diagnosed with congenital kyphoscoliosis and had frequent visits to the orthopedic clinic because of pain. We performed a spirometry, which showed a restrictive pattern. The child´s head was leaning forward, with a stiff back and curved shoulders. Cobb´s angle was measured which was approximately 58°. The child was screened for cardiac anomalies and genitourinary abnormalities but was not suffering from any of the other ailments. She was advised chest physiotherapy and breathing exercises, but looking at the progressive nature of the vertebral defect, she was referred to a spine surgeon for corrective surgery.

**Figure 1:**
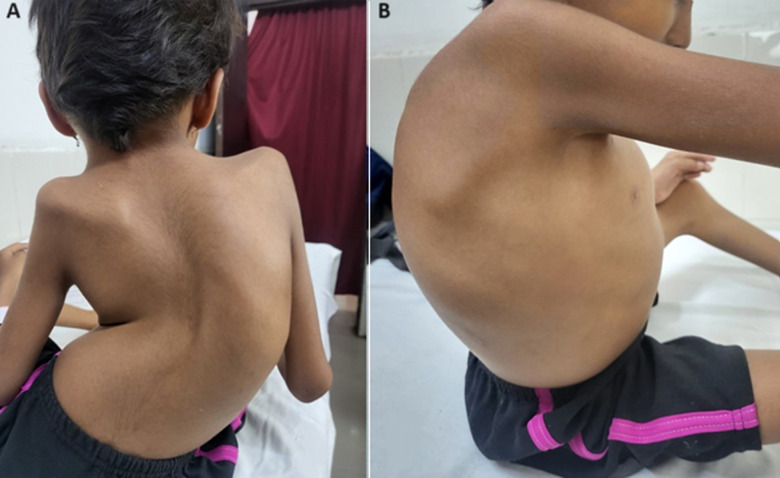
A) severe scoliosis; B) kyphosis

